# Editorial: Mechanisms and therapeutic opportunities of T-cell impairment in cancer immunity and immunotherapy

**DOI:** 10.3389/fimmu.2026.1795868

**Published:** 2026-02-12

**Authors:** Guillaume Beyrend-Frizon

**Affiliations:** Visualyte, Olley, France

**Keywords:** apoptosis, ICOS, B-ALL, B-cell acute lymphoblastic leukemia, breast cancer, BTG3, CD107a, CD276

## Introduction

The success of immune checkpoint inhibitors (ICIs) targeting PD-1/PD-L1 and CTLA-4 has revolutionized cancer treatment, yet our understanding of immune checkpoint biology continues to evolve in unexpected ways. The traditional paradigm positions immune checkpoints as inhibitory molecules that tumors exploit to evade immune destruction—a framework that, while therapeutically productive, may oversimplify the nuanced roles these molecules play in anti-tumor immunity. This Research Topic challenges conventional assumptions about immune checkpoint function and T cell biology in cancer, revealing that molecules traditionally viewed as markers of dysfunction can, paradoxically, identify highly functional cytotoxic T cells under specific contexts.

The studies assembled here bring together complementary investigations spanning multiple cancer types—B-cell acute lymphoblastic leukemia (B-ALL), gastric cancer, liver metastases, head and neck squamous cell carcinoma (HNSCC), and breast cancer—employing cutting-edge methodologies including single-cell RNA sequencing (scRNA-seq), spatial transcriptomics, and machine learning approaches. Collectively, these contributions illuminate three interconnected themes: the context-dependent roles of immune checkpoints, the complex interplay between programmed cell death and immune evasion, and the transformative potential of advanced computational methods in deciphering tumor immunity.

## B7-H3 and LAG3: from exhaustion markers to functional identifiers

Perhaps the most striking conceptual shift emerges from the work of Tamura et al., who demonstrate that co-expression of B7-H3 (CD276) and LAG3—molecules conventionally associated with T cell exhaustion—actually identifies highly functional CD4+ cytotoxic T lymphocytes (CTLs) in humans. Using an Epstein-Barr virus (EBV)-driven model system, the authors show that B7-H3+LAG3+ CD4+ T cells exhibit markedly enhanced cytotoxic capacity compared to B7-H3−LAG3− counterparts, characterized by elevated expression of granzyme B, perforin, CD107a (a degranulation marker), and multiple killing pathways including FasL, TRAIL, and IFN-γ.

Importantly, this co-expression pattern is target-dependent—induced by interaction with antigen-presenting cells but not by anti-CD3/CD28 stimulation alone—and specific to CD4+ T cells, as B7-H3+LAG3+ CD8+ T cells were scarcely detected. The clinical relevance was validated in bone marrow samples from pediatric B-ALL patients, where B7-H3+LAG3+ CD4+ T cells expanded ex vivo and demonstrated superior cytotoxic potential. These findings fundamentally challenge the binary classification of immune checkpoints as simply “inhibitory” or “stimulatory.”

This paradigm is further complicated by observations across other studies in this Research Topic. In the HNSCC study by Chen et al., LAG3 expression correlated with T cell dysfunction in some contexts, while B7-H3 was associated with tumor progression when expressed on malignant cells. Similarly, in the breast cancer study by Xie et al., LAG3 appears among the canonical exhaustion markers (alongside PD-1, CTLA-4, TIM-3, and TIGIT) associated with impaired CD8+ T cell function. The gastric cancer review by Luo et al. discusses LAG3 upregulation as a consequence of chronic antigenic stimulation leading to mitochondrial dysfunction and exhaustion.

How do we reconcile these seemingly contradictory observations? The answer likely resides in cellular context, co-expression patterns, and the specific immunological microenvironment. B7-H3 and LAG3 may serve as “rheostats” rather than simple on/off switches—their functional consequences determined by the constellation of other receptors expressed, the activation state of the cell, and the nature of the antigenic stimulus. The Tamura study suggests that in CD4+ CTLs responding to MHC class II-restricted antigens, these molecules may mark cells at peak functional capacity rather than terminal exhaustion. This interpretation aligns with recent reconceptualizations of exhaustion as a spectrum rather than a discrete state, with different checkpoint molecule combinations defining functionally distinct subpopulations.

## Cellular heterogeneity and programmed cell death in the tumor microenvironment

The tumor microenvironment (TME) represents a complex ecosystem where immune surveillance, tumor evasion, and programmed cell death pathways intersect. The comprehensive analysis by Chen et al. employing integrated single-cell, spatial, and bulk RNA sequencing in HNSCC provides a particularly illuminating perspective on these dynamics.

Using scRNA-seq data from over 326,000 cells across five HNSCC subtypes, Chen et al. constructed a single-cell atlas revealing that T cells—not malignant cells—exhibited the highest levels of cell death. Among eighteen distinct cell death modalities (CDMs) analyzed, including apoptosis, necroptosis, ferroptosis, pyroptosis, cuproptosis, and others, functional T cells demonstrated elevated cuproptosis and alkaliptosis, while malignant cells showed relatively suppressed cell death programs. Strikingly, HPV infection attenuated cell death in malignant cells, potentially explaining the different clinical behavior of HPV-positive versus HPV-negative HNSCC.

Through machine learning integration of ten algorithms generating 100 predictive models, the authors developed a consensus cell death-related signature (CDRscore) based on ten prognostic genes (MRPL10, DDX19A, NDFIP1, PCMT1, HPRT1, SLC2A3, EFNB2, HK1, BTG3, MAP2K7). Patients with high CDRscore manifested worse overall survival, elevated epithelial-mesenchymal transition, enhanced TGF-β signaling, increased hypoxia, higher expression of T cell exhaustion markers, and greater TP53 mutation frequency. Spatial transcriptomics validation in laryngeal squamous cell carcinoma (LSCC) confirmed that high-risk spots colocalized with TGF-β signaling and proliferating malignant cells, while showing negative correlation with TCR signaling and positive association with LAG3 expression.

These findings complement the observations in other tumor types. In the liver metastasis review by Liu et al., the immunotolerant hepatic microenvironment promotes metastatic colonization through multiple cell death evasion mechanisms, including Kupffer cell polarization, neutrophil extracellular trap (NET) formation, and metabolic constraints (glucose depletion, lactate accumulation) that suppress NK and CD8+ T cell function. The gastric cancer manuscript by Luo et al. similarly emphasizes metabolic reprogramming—particularly aerobic glycolysis leading to glucose depletion and lactic acid accumulation—as central to CD8+ T cell exhaustion.

The breast cancer exhaustion study by Xie et al. adds further mechanistic layers, describing how tumor-associated macrophages (TAMs), particularly M2-polarized macrophages, promote CD8+ T cell exhaustion through ICAM-1 interactions, while tumor-derived extracellular vesicles (TEVs) deliver active TGF-β type II receptor to CD8+ T cells, inducing SMAD3 activation and exhaustion. Intriguingly, the exosomal pathway emerges as a critical mechanism whereby tumors systemically suppress anti-tumor immunity, with exosomal PD-L1 (exoPD-L1) binding to PD-1 on CD8+ T cells at metastatic sites.

## The transformative role of advanced technologies and artificial intelligence

A remarkable feature of the studies in this Research Topic is the sophisticated deployment of emerging technologies that are reshaping our understanding of tumor immunology. The integration of single-cell RNA sequencing, spatial transcriptomics, and machine learning represents a powerful synergy that transcends what any single approach could achieve.

Chen et al.’s machine learning framework exemplifies this paradigm shift. By integrating ten algorithms—including random survival forest (RSF), elastic network (Enet), LASSO, Ridge, stepwise Cox, CoxBoost, partial least squares regression for Cox (plsRcox), supervised principal components (SuperPC), generalized boosted regression modeling (GBM), and survival support vector machine (survival-SVM)—the authors generated 100 algorithm combinations, ultimately identifying the optimal model (RSF combined with superPC) based on the highest average C-index across validation datasets. This integrative approach yielded a prognostic signature with superior performance to individual clinical variables including TNM stage, tumor mutation burden (TMB), and microsatellite instability (MSI).

The spatial transcriptomics component proved particularly valuable, enabling the authors to validate computational predictions *in situ*. By examining the spatial colocalization of high CDRscore spots with TGF-β signaling and proliferating malignant cells—specifically at the interface between malignant cells and immune cells—the study demonstrates how computational predictions derived from bulk and single-cell data can be ground-truthed in their native spatial context.

This democratization of complex analytical approaches through AI-driven tools has profound implications. As the lead author noted in the research context, AI “democratizes access to complex analyses (big data, coding) previously limited to specialists.” This accessibility enables clinician-scientists to perform sophisticated transcriptomic and prognostic analyses that previously required extensive bioinformatics expertise. The HNSCC study leveraged publicly available datasets from TCGA and multiple GEO repositories (GSE103322, GSE148673, GSE150321, GSE162025, GSE172577, GSE181919, GSE41613, GSE65858), demonstrating how AI-powered integration of heterogeneous data sources can generate clinically actionable insights.

Single-cell technologies similarly reveal tumor immune heterogeneity invisible to bulk sequencing. Tamura et al.‘s comparative transcriptomic analysis of functional versus dysfunctional CD4+ CTLs identified B7-H3 and LAG3 from 156 differentially expressed genes, filtering through 51 membrane protein-encoding genes to ultimately focus on nine candidates—an analytical pipeline that would be intractable without computational support. The breast cancer study by Xie et al. employed single-cell profiling to demonstrate that dysfunctional PD-1+ CD8+ T cells are enriched with EOMES and nuclear LSD1, revealing epigenetic mechanisms of exhaustion.

## Therapeutic implications: beyond checkpoint blockade

The insights from this Research Topic carry significant therapeutic implications that extend beyond conventional checkpoint blockade strategies.

First, the Tamura study suggests that B7-H3 and LAG3 co-expression could serve as biomarkers for identifying tumor-reactive CD4+ CTLs in adoptive cell therapy (ACT) approaches. Given that CD4+ CTLs demonstrated prolonged proliferation and sustained cytotoxic activity compared to CD8+ CTLs, selectively expanding or engineering B7-H3+LAG3+ CD4+ T cells might enhance therapeutic efficacy, particularly in MHC class II-expressing tumors like B-ALL, melanoma, and certain carcinomas. However, the context-dependency of these markers necessitates careful validation in each tumor type.

Second, the CDRscore model developed by Chen et al. offers a prognostic tool for stratifying HNSCC patients who might benefit from intensified therapy or novel interventions targeting cell death pathways. The colocalization of high-risk scores with TGF-β signaling suggests that combining TGF-β inhibition with immunotherapy could prove particularly effective in high-CDRscore patients. Indeed, several manuscripts highlight TGF-β as a central mediator of immunosuppression, promoting T regulatory cell expansion, M2 macrophage polarization, and direct T cell exhaustion.

Third, the liver metastasis review by Liu et al. emphasizes targeting the myeloid compartment—particularly tumor-associated macrophages (TAMs), myeloid-derived suppressor cells (MDSCs), and neutrophils—as a complementary strategy to checkpoint blockade. Clinical trials combining CSF1R inhibitors (pexidartinib) with PD-L1 blockade (durvalumab), CCR2 antagonists (CCX872) with chemotherapy, and CD40 agonists with chemotherapy ± nivolumab have shown promising early results, though compensatory mechanisms (GM-CSF/G-CSF bypass, recruitment of alternative immunosuppressive cells) remain challenging.

Fourth, the metabolic reprogramming axis emerges as an attractive therapeutic target across multiple tumor types. Metformin, highlighted in the breast cancer manuscript, reduces tumor hypoxia and PD-L1 expression while enhancing T cell function, positioning it as a potential adjunct to ICIs in refractory tumors. Similarly, targeting ferroptosis (through SLC7A11 inhibition) or cuproptosis pathways may selectively eliminate tumor cells while sparing T cells, as suggested by differential cell death patterns observed in the HNSCC study.

Finally, the exosomal pathway represents an emerging therapeutic frontier. The breast cancer study describes how tumor-derived exosomes suppress CD8+ T cell glycolysis via AKT-mTOR-dependent mechanisms and deliver immunosuppressive cargo including PD-L1 and TGF-β receptor to distant sites. Targeting exosome biogenesis, release, or uptake could therefore provide systemic immunomodulation complementing local checkpoint blockade.

## Perspectives and future directions

The studies assembled in this Research Topic collectively advance our understanding of cancer immunity while raising new questions that merit investigation.

Refining checkpoint biology: The B7-H3/LAG3 paradox underscores the need for more nuanced models of checkpoint function. Rather than viewing these molecules as uniformly inhibitory, we should investigate the signaling networks, co-receptors, and transcriptional programs that determine whether checkpoint engagement promotes or restrains T cell function. The absence of validated receptors for B7-H3 (with TLT-2 and IL20RA remaining controversial) particularly hampers mechanistic understanding.

Integrating multi-omic layers: While this Research Topic leverages transcriptomics extensively, integrating proteomics, metabolomics, and epigenomics would provide a more complete picture of T cell states. The breast cancer study’s findings on epigenetic regulation (LSD1, TWIST1, BET proteins, m6A modifications) hint at complex post-transcriptional and post-translational regulatory layers that single-cell RNA sequencing alone cannot fully capture.

Longitudinal studies: Most analyses represent cross-sectional snapshots, yet T cell exhaustion is a dynamic, evolving process. Longitudinal studies tracking individual patients through treatment could reveal how checkpoint expression patterns and cell death programs shift in response to therapy, potentially identifying early biomarkers of resistance or response.

Causal validation: Computational predictions require experimental validation. While spatial transcriptomics provides anatomical validation, functional validation through genetic perturbation (CRISPR screens, CAR-T engineering) would establish causal relationships between checkpoint expression and cytotoxic function.

Clinical translation: The CDRscore model and B7-H3/LAG3 biomarkers require prospective validation in clinical trials. Retrospective analysis of existing ICI trial cohorts could determine whether these signatures predict treatment response, while prospective biomarker-driven trials could test therapeutic hypotheses.

Expanding tumor coverage: The studies presented here focus primarily on solid tumors and B-ALL. Extending these analyses to other hematological malignancies and tumor types would reveal whether the observed patterns represent universal principles or context-specific phenomena.

## Conclusion

This Research Topic collectively challenges us to rethink fundamental assumptions about immune checkpoint biology and T cell dysfunction in cancer. The demonstration that B7-H3 and LAG3—canonical exhaustion markers—can identify highly functional CD4+ cytotoxic T cells exemplifies how context shapes function. The integration of single-cell technologies, spatial transcriptomics, and machine learning provides unprecedented resolution for dissecting tumor immune ecosystems, while highlighting the complex interplay between programmed cell death, metabolic constraints, and immune evasion.

As we move beyond first-generation checkpoint inhibitors toward more sophisticated immunotherapies, these insights offer a roadmap for rationally designed combination strategies targeting multiple immunosuppressive mechanisms simultaneously. The democratization of AI-powered analytical tools promises to accelerate this translation, enabling broader participation in cutting-edge immuno-oncology research. Ultimately, by embracing the complexity and context-dependency of immune checkpoint biology, we may unlock more effective, personalized approaches to cancer immunotherapy.

